# Se-duction is not sex-duction: Desexualizing and de-feminizing hysteria

**DOI:** 10.3389/fpsyg.2022.963117

**Published:** 2022-09-23

**Authors:** Milena Mancini, Martina Scudiero, Silvio Mignogna, Valentina Urso, Giovanni Stanghellini

**Affiliations:** ^1^Department of Psychological, Humanistic, and Territorial Sciences, University “G. D’Annunzio”, Chieti, Italy; ^2^Department of Health Sciences, University of Florence, Florence, Italy; ^3^Centro de Estudios de Fenomenologia y Psiquiatrías – Diego Portales’ University, Santiago, Chile

**Keywords:** hysteria, desexualizing, de-feminizing, visibility, phenomenological psychopathology, subjective experience

## Abstract

The psychopathological analysis of hysteria is a victim of narrow conceptualizations. Among these is the inscription of hysteria in the feminine sphere, about body and sexuality, which incentivized conceptual reductionism. Hysteria has been mainly considered a gendered pathology, almost exclusively female, and it has been associated with cultural and/or religious features over time rather than treated as a psychopathological world. Further, hysteria has been dominated by conceptual inaccuracies and indecision, not only in terms of clinical features but also in terms of its definition. For this reason, it seems necessary to “undress” hysteria from this feminization, sexualization, and corporealization with which it has been abundantly clothed over the years. “Undressing” hysteria will make possible a reconfiguring and deconstructing of the explanatory-causal model of Charcot and Freud. However, if we take out this cultural heritage, the stigma accompanying this diagnosis, and the weight of the enormous historical tradition that hysteria carries, the world of hysteria continues to constitute a domain full of complexity and nosographic challenges. Hysteria has been considered a sum of psychological behaviors and states illustrated by drama, mystery, or falsity. The difficulty in understanding the multiple somatic manifestations which characterize this clinical condition created several controversies and much confusion. In the current nosography, the personological component of hysteria has been separated from its symptomatic manifestation, in the Histrionic Personality Disorder and Conversion Disorder categories, respectively. This segmentation by descriptive nosography does contribute to a unitary understanding of the phenomenon and, consequently, of daily clinical practice. Clinical complexity can be grasped and deciphered only if the symptom is inscribed in the patient’s lifeworld and his/her subjective life history. Clinical practice is thus thought of in terms of a structural aggregation of a homogeneous set of phenomena, together constituting a specific way of being in the world. The starting point of this article is the evident modalities characterizing this life-world, taking care not to confuse the point of origin with the point of expression.

## Introduction: A short history of hysteria as a gender disorder

There is a clinical condition that throughout history has been responsible for much of the stigma associated with feminine corporeality and sexuality, known as “female hysteria” (Tasca et al., 2012; Ussher, 2013; Hooper et al., 2019; Gallic, 2021). Before its classification as a mental disorder, hysteria was considered a gender-specific physical disorder affecting individuals with a uterus. The first evidence of hysteria can be found in ancient Egyptian and Greek cultures. Hippocrates (459–376 BC), who first introduced the clinical use of the term “hysteria,” believed that the uterus (*hysteron* means “uterus” in ancient Greek) could migrate around the body, pressuring other internal organs and causing several diseases (Sigerist, 1951; Trimble and Reynolds, 2016). According to ancient physicians, sexual deprivation was frequently the cause of hysteria (Williams, 2022). Symptoms associated with hysteria included swollen abdomen, suffocating angina or dyspnea, dysphagia, cold extremities, tears/laughter oscitation, pandiculation, delirium, and irregular heart rate. The treatment of hysteria involved the repositioning of the uterus through various techniques.

From the advent of Christianity until the Middle Ages, hysteria was associated with phenomena such as witchcraft and demonic possession. The mainstream view of those times conceived the woman as a physically and theologically inferior being, i.e., a “failed man,” an idea with its roots in the Aristotelian concept of male superiority. Thus, women were considered sinful and defective creatures (the Latin term *foemina* means “who has less faith”). From the 13th century onwards, many manifestations of mental illness were seen as the consequences of bonds with forces of evil. At that time, the gold-standard treatment to heal “hysterical” women was an exorcism. In early Christianity, exorcism was considered a cure, but by the late Middle Ages, it had become a punishment, and hysteria was confused with witchcraft (Alexander and Selesnick, 1975).

Paracelsus (1493–1,541) considered hysteria a chorea lasciva, a lecherous or lustful dance, patently connecting it to femininity and sexuality. During the Enlightenment, sorcery becomes a matter of medical care. In the Encyclopédie, sorcery was viewed as a ridiculous activity and mental illness began to be framed within a scientific perspective. Hysteria itself was described as one of the most complicated diseases (Diderot and D'Alembert, 1968).

Throughout the 18th and 19th centuries, hysteria was one of the most commonly diagnosed “disorders.” In modern medicine, hysteria was first accurately described in 1880 by Jean-Martin Charcot (1825–1893). Freud attended Charcot’s lessons at the Paris hospital La Salpetrière. Building on and extending Charcot’s theories, he hypothesized that hysteria was the outcome of a “psychological scar,” triggered by trauma or repression, rather than the consequence of a physical injury affecting the brain (Charcot, 1998; Leoni, 2008). The highlighting of the psychic etiology of hysteria goes hand in hand with the main discoveries of psychoanalysis, i.e., the unconscious, conflict, trauma, defenses, transference, and identification (Laplanche et al., 2018). Sexuality was placed center stage. The conflict unleashed against certain sexual impulses was considered the basis of hysteria and all other neuroses. At first, Freud believed that hysteria could be explained as the effect of missing abreaction of affective charges related to the memory of a traumatic event in childhood, experiences of sexual abuse or incest in particular. Later, Freud considered that this seduction had never taken place in reality but only in the imagination, and reduced the emphasis on abuse in childhood by focussing on the sexual fantasies of the child (Lingiardi and McWilliams, 2017).

The ‘father of psychoanalysis’ believed that women developed hysteria because they could not recognize and overcome their castration complex. From time to time, researchers of medical history have evidenced that hysteria was a way to pathologize “everything that men found mysterious or unmanageable in women” (Devereux, 2014). Treatments for this condition ranged from pelvic or uterine massages, to forced rest, or even marriage. In severe cases, a hysterectomy was performed.

From a gender perspective, hysteria has essentially been conceived as the medical justification for men’s dominance over society and medicine and a synonym supported by “over-emotional” or “unbalanced,” which were deemed to be characteristics of the female gender. Simone De Beauvoir (1908–1986) wrote of the “complex” female nature, which according to many was guided by hormones, mysterious instincts, and repressed desires: “The ovule has sometimes been likened to immanence, the sperm to transcendence” (De Beauvoir, 1989). Similar gendered and sexualized stereotypes include the belief that women should be obedient, passive, moderated, and sexually inhibited. It is not a coincidence that most prescriptions for hysteria involved regular marital sex, marriage, or pregnancy and childbirth: all accomplishments for a culturally framed “proper” woman.

Borrowing Michel Foucault’s (1926–1984) expression, society has always influenced psychiatry’s judgment about normality or disease (Foucault, 1961). This idea is tangible in hysteria more than in any other diagnostic category (Tasca et al., 2012). In the second half of the 20th century, a “decrease” in hysteria was recorded in Western societies. In contrast, studies focused on non-Western countries demonstrated that during the same period, hysteria, as one of the somatic ways of expressing emotional distress, remained a prominent condition among psychiatric patients (Leff, 1981). Up to a few decades ago, hysteria-like phenomena were also reported in Southern Italy by cultural anthropologist Ernesto De Martino (1908–1965). He studied the phenomenon called “tarantism.” In southern Apulia, people believed that the bite of a particular species of spider (“tarantula”) could cause a psycho-physical disorder – a form of hysterical neurosis manifesting with body spasms and convulsions. Its “treatment” consisted of music therapy and dance sessions (De Martino, 2015). Moreover, tarantism was an exclusively female disorder.

Hysteria has been used as an instrument of power to sanction women’s perceived intellectual, physical, and moral inferiority. This controversial clinical condition was used to justify the confinement, control, and pathologization of women. Today, this attitude has apparently been overcome. However, it has taken on new forms. For example, medical operators are more inclined to describe the pain of patients with adjectives such as “emotional, psychogenic, hysterical or hypersensitive” when those patients are women (Zahang et al., 2021). Hysteria was officially removed from DSM after being used as an umbrella term to encompass numerous different symptoms, reinforcing injurious stereotypes about sex and gender. Recent nosography of hysteria was rather convoluted. In DSM-I hysteria was designated the name “conversion disorder” and in DSM-II it was termed “hysterical neurosis” (American Psychiatric Association, 1952, 1968). Starting from DSM-III, it was included among “somatoform disorders” (American Psychiatric Association, 1983). More recently, the personological (trait) component of hysteria has been separated from its symptomatic (state) manifestation, into Histrionic Personality Disorder and Conversion Disorder, respectively. The latter is also known as Functional Neurological Disorder (American Psychiatric Association, 2014). Other aspects of hysteria are located in the category of Dissociative Disorder. In this article, we focus on the histrionic personality, not on other aspects of “hysterical neuroses.” Unfortunately, despite its exclusion from official psychiatric nosography, the stigma associated with this peculiar condition persists nowadays, both in clinical and non-clinical contexts.

## The body in between sexuality and seduction

The body represents the most investigated dimension in the world of hysteria (Didi-Huberman, 2004; Stanghellini and Mancini, 2017, 2018). Freud (1886–1895) believed that hysteria was first and foremost a diagnosis of the body and hysterical symptoms, based on the expressive power of the body, were its “symbolic dimension,” its theatricality, and its metaphorical significance. This almost exclusively bodily conceptualization of the hysterical symptoms is coherently linked to the question of femininity and sexuality. Moving in this reductionist direction, the hysteric person is a woman, particularly a woman who attempts to name herself as such through the exhibition of her body. Here, the body is the locus of the hysterical symptoms since the body is allowed to name the un-nameable (Bollas, 2001).

The body no longer represents a way to be in a dialogical relationship with the other person (Binswanger, 1942; Richin, 1989) but is exhausted in provoking an emotional reaction in the other. Stripped of its dialogical connotation, it is clothed in mannerisms, poses, and seductiveness. Its mere purpose is to seduce the other in the etymological sense (*se ducere*) of leading the other to oneself and trapping them in the kind of bond that makes any sort of dialogue impossible. The other becomes a mere spectator of a scene in which the center is occupied by the hysterical person and his/her extreme intensification of attitudes and poses. The hysterical person’s body is an instrument, a mere means to capture the gaze of the other. It is an “instrumentalized” body (Blankenburg, 1998), mutable in its intense and hyper-expressive choreography. It is a spectacular representation, a performance worthy of being immortalized and remembered.

Nevertheless, if seduction – attracting the other’s gaze – is the aim of the person with hysteria, sexuality is only one of the possible ways to seduce the other, just one of the manifold trajectories and not the core of the hysterical world. Sexuality is a means, not an end, in the world of the hysterical person. Seduction is not meant to catch the other on a sexual level for sexual enjoyment or sexual satisfaction. It can operate in several other domains, e.g., exhibiting one’s intellectual abilities, intensifying one’s suffering, or theatrically showing one’s generosity.

Typical of the hysterical condition is *impersonation* (Sartre, 1943), i.e., role-playing or play-acting, embodying a given role in a self-deceptive manner that is unconscious and involuntary. Impersonation can operate in different domains. For instance, the person with hysteria can embody the role of a person affected by a physical illness, identifying with his/her symptoms as a way to gain visibility. The identification with a person affected by a physical illness is part of so-called somatoform disorders, the cause of which can be traced back to an incapacity to “mentalize” emotional distress (PDM) and the need to gain attention by playing the role of the sick person. The fact that the hysterical person does not do it consciously and voluntarily suggests that (s)he, to lie to others, is forced to lie to themselves. This is not a deliberate fiction but an extreme self-deceptive attempt to exist. For the hysterical person, being perceived, seen, and heard is an indispensable condition for being in general: *esse est percipi*, being is being perceived.

Seduction is thus a means to recognition (Stanghellini, 2016). We can argue that the hysterical person experiences a “manque d’être” (Sartre, 1943) – a lack of being. Experiencing a constant feeling of hypo-sufficiency, (s)he depends on the other to obtain a consistent sense of selfhood and identity. From this perspective, seduction is instrumental for recognition, and recognition is instrumental for gaining a sense of selfhood: when (s)he manages to catch the other’s gaze, the hysterical person feels that he/she exists, and his/her feeling of inadequacy and insufficiency vanishes. The logic of the hysterical world follows the motto *seduco ergo sum*. Seduction in the hysterical person’s world is the visual power to be recognized by the Other, a device of self-recognition whose aim is to construct a sense of selfhood and defeat hypo-sufficiency. The hysterical person aims to achieve a “consistency of being” in which (s)he can experience the gratifying feeling of “being special” to the other. The aim is not to dominate the other, but rather to bring the other as close as possible to oneself to feel visible. Putting the hysterical persons’ need for recognition center stage strips them of the gender and sexual aspect with which they have been clothed over time.

Two modes of seduction can be preliminarily traced from here. The first is the intensification of one’s attitudes and feelings, defined by Charbonneau (2007) as “figurality.” The other is placed on a pedestal, and every his/her attitude, word, and posture have the aim of being admired by the idealized other. The second mode of capturing the other’s gaze is *via* self-victimization and infantilism with the aim of being cared for by the other. Both modes have in common the aim of keeping the other available and attentive, capturing and keeping their gaze fixed on oneself as compensation for one’s feelings of hypo-sufficiency.

## Visibility as the core of seduction

The dismantling of the predominant idea of sexuality in the hysterical world and the primacy of seduction brings out another characteristic: visibility. Throughout history, hysteria has been sorrow reinvented as spectacle and an image. Everything that produces passion in the soul pushes the body to some form of action or expression. Hysteria is nothing more than a sentimental experience: soul affections become bodily catastrophes, an enigmatic and violent manifestation of otherwise invisible feelings. The symptom (“What do you feel?”) becomes a visible sign, and the diagnosis becomes a skillful interpretation of what is visible when armed with photography. Photography brought hysteria center stage at the end of the 19th century. In Charcot’s time, hysterical patients were shown to an audience of doctors and photographed. This indulging in their gaze allowed the persons with hysteria not to be relegated and forgotten in the pavilion of the “Incurables.” The only way they could avoid being neglected was to comply with the doctors’ requests. The seductive character restored their visibility and centrality by allowing them not merely to define themselves, but to save their lives from oblivion.

The cultural history of hysteria shows that one cannot know hysteria before photographing it. Meticulous visual observation was the origin of all Charcot’s discoveries. An image will always say more than the best description. Charcot’s use of photography aimed to translate and fix in an “alphabet of the visible” the “states of the body” (Didi-Huberman, 2020, pp. 54–55). Photography has an oracular power to reveal what Benjamin called the “optical unconscious,” the unconscious that is revealed only through careful inspection of the image. The hysterical person’s observable signs make her/his secret an almost visible pose. Nevertheless, this sign could only be photographed if the person posed, that is, if she remained motionless in front of the lens, sometimes for hours. This exaggerated the visibility of the hysterical signs. Charcot gave new life to hysteric women, but at the same time he relegated hysteria to the realm of fabrication, or as Didi-Huberman (2020) put it, the realm of lies. Photography was a “frenzied and almost ignoble” attempt (*ibidem*, p. 46), an exaggerated “extension of the evidence” (*ibidem*, p. 253), and thus a falsification (*ibidem*, p. 35). Through photography, and thus the visual cataloging of symptoms into “iconic narratives” or “pictures’ devoid of context, the ideal of an absolute (clinical and not only) eye” was realized (*ibidem*, p. 59).

Beyond Charcot’s intentions, the relevant aspect here is precisely this gallery or catwalk of pain and exhibition. In a kind of voyeuristic game, the most compliant of the models, she who would otherwise have been imprisoned in the pavilion of invisibility, where all gaze was denied, exaggerated her dance of pain according to the desire of the gaze of the doctors and other voyeurs. Being on the photographic set of the Salpêtrière allowed these women both to be visible and unforgettable in their exasperated poses - and to be somebody, as was the case with Mademoiselle Augustine, the star, the masterpiece of Charcot’s theatre of hysteria.

To be(come) somebody, you need to be(come) visible. Visibility is the core of seduction. In order to seduce, one must be visible, or better, hyper-visible to capture and hold the gaze of the other. Charbonneau (2007) emphasizes the character of centrality, which could be considered a way of being visible here. The hysterical person always needs another, therefore, a gaze to show oneself to. The Other is pure gaze: he/she is a compliant witness to a hysterical person’s performance. As Jaspers (1913) states, the hysterical world is characterized by “[...] desire to appear, both for oneself and for others [...].” The hysterical person is dependent on other people’s meanings and values, or what (s)he considers to be such. Furthermore, the hysterical person is not satisfied with being seen but wants to be memorable or impressive. To achieve the goal of visibility, the hysterical person embodies a caricatured version of different human types, by exaggerating and intensifying them. (S)he is not actually a person but a figure (hence figurality) and gets trapped both in a universe of types to represent and through which to attract the others’ gaze, and in postures and attitudes to impress others. This mode corresponds to what Charbonneau (2007) called typification, i.e., “embodying a totality in a figurative way.”

## Beyond gender: Degenderizing the hysteric world

Undressing the world of hysterical persons of sexuality and dressing it in seduction, on one hand, and visibility, on the other, leads us toward deconstructing hysteria as a gender pathology belonging to the feminine. The attempt to de-feminize hysteria is not new. The notion of male hysteria was initially connected to the post-traumatic disorder known as railway spine (Lerner, 2003); later, it became associated with war neurosis to prevent labeling soldiers with the “feminizing” label of hysteria (Showalter, 2020). Charcot himself seemed to have accepted the existence of male hysteria and distinguished it from female hysteria by linking it to traumatic shock rather than sexuality or emotional distress. Despite Charcot’s attempt to recognize male hysteria, gender stereotypes were prevalent in his thinking (Lerner, 2003). Freud, in his analysis, argued that trauma was the cause of both male and female hysteria and that both had reason to exist.

Although in different ways, the hysterical way of being in the world concerns both women and men, as both are concerned with the issues of lack of being, seduction, and visibility. Certainly, seduction and visibility follow different trajectories in the female and male genders, but in both cases, the aim is the same: to engage the gaze of the other in order to overcome one’s lack of being and thus define oneself. Relegating the others to spectators of the hysterical figurality does not allow for dialogue with them. Reciprocity is overwhelmed by visibility and “we-ness” by centrality.

The hysterical persons constantly live in the abolition of the private sphere. In other words, they live in a kind of overflow in the space in between oneself and others that is no longer inter-subjective but hyper-subjective, i.e., saturated with the subjectivity of the hysterical person and his/her centrality and figurality, relegating the other to the position of a mere spectator. The relational space, therefore, is a hypertrophically centralized space that is no longer a space to dialogue with the other, in which “I” and “Thou” can know and each other. Approaching the other would mean not only creating the basis for a relationship, but also decentralizing and renouncing emotional intensification, exchanging one’s own desires with the other, and becoming intimate partners. From this angle, hysteria is a pathology of the inter-subjective space, in which there is a precipitous “reversal toward the center” (Charbonneau, 2007).

## Conclusion: Lack of being, seduction, and visibility

As a way of being in the world, and not of being a woman, hysteria cannot in any way be polarized to the feminine world (see Esposito and Stanghellini, in press). If hysteria is not a gender pathology, then what is it a pathology of?

In this article we have discussed how at the core of the hysteric condition there is a lack of being, without explaining what we mean by this. This term is derived from Sartre, 1943 ontological analyses of the human condition. According to Sartre, the human condition is lacking because it is originally constituted as the fall of the Self from being in itself to being for itself, a form of consciousness that always feels separated from itself and contingent in its radical lack of foundation. In a nutshell, rather than feeling “at one” with oneself, coinciding with one’s center, one feels like a spectator, looking at oneself from outside.

This essential feature of the human condition is epitomized in the psychopathological symptom called depersonalization, ie., a human being is an essentially and radically depersonalized form of being. A concise clinical vignette of a patient of one of us can help to summarize this phenomenon: “I feel distant from myself, as if I were not myself or in myself. As if I did not coincide with myself. I feel miserable because I cannot feel myself. I have not a basis on which I can build myself, my projects, my relationships.”

From the angle of depersonalization, a lack of being can sometimes entail another psychopathological phenomenon called “autoscopy,” seeing oneself from without. The narrative goes like this: “Sometimes, it is as if I were watching myself from without when I am doing something. Especially when I am deeply stressed, or perhaps when this happens as a consequence, I feel extremely stressed and anxious.” Autoscopy is indeed a symptom listed for dissociative disorders, and a feature of hysteria. The patient’s narrative continues: “Do not you see how mechanical, how unnatural, are my gestures? I feel like a mannequin. I realize how my movements are baroque, exaggerated, one pose after the other. As if I were cut in wood instead of being made of flesh.” This feeling of inauthenticity and a kind of manneristic behavior are also symptoms of hysteria.

Lack of being is an unbearable feeling which urges a remedy, a distressing phenomenon that may kindle an attempt at self-healing or trigger a defense mechanism. From this angle, seduction can be seen as a means to compensate for the lack of being – one of many available, involuntary, and almost unconscious, compensating strategies to cope with lack of being, yet the one specific to the condition we call “hysteria.” Minkowski (1927) developed the concept of “phenomenological compensation” and adopted it to present a dynamic view of the pathogenesis of schizophrenic symptomatology. This concept can be helpful to make sense of how persons with hysteria deal with lack of being. Seduction may be regarded as a secondary, reactive, or compensatory attempt to cover up for the primary lack of being. Persons diagnosed with hysteria try to compensate for their lack of being by seducing others, that is, capturing the attention of the other and using the other as a prosthesis in order to achieve a better “rooting” into themselves. The others’ gaze is instrumental in reducing their uncanny feeling of lack of being.

Seduction, in its own turn, needs visibility. “I am afraid I became addicted to others, to being at the center of their attention. Not all others: those that are important to me. I always fall in love with those I call “the boss”: the owner of the gym where I go for training, an older colleague, the doctor. Being at the center of their world gives me the feeling of being in touch with myself. However, it lasts very little time. Furthermore, in the end, I am back once more with this lack of myself.”

How to become visible to others? One way is erotic seduction, *via*, for instance, the sexualization of relationships, as was the case during the long history of hysteria, culminating in Charcot’s theatre with his hysterical vedettes. However, as we have seen throughout this article, there are several other ways to capture the other’s gaze, including self-victimization, infantilism, intellect brightness, and hyper-generosity. These are all tactics to achieve recognition from the other. In this vein, as with the entire psychodynamic and phenomenological understanding of psychopathological signs and symptoms (Stanghellini and Mancini, 2017, 2018), these are, rather than ready-made outputs of a deranged brain or mind, the self-healing attempts of a person struggling against an intolerable and uncanny experience like lack of being.

In this context, the clinical complexity of hysterical signs and symptoms can be grasped and deciphered as inscribed in the patients’ life-world and in her/his life history. This is a specific kind of psychopathological condition in which a lack of coenaesthetic contact with oneself is compensated by seducing the other, gaining some sort of visibility by impressing them with theatrical, dramatic, or intensified poses (Stanghellini, 2019; Stanghellini et al., 2019; Mancini and Esposito, 2021).

Understanding hysteria as an anthropological unit, beyond the feminine and the masculine, is the route to accessing meaning and bringing down the last bastion: that hysteria is female.

To avoid generalizations and misunderstandings, we would like to add the following remarks before concluding our article. First, seduction – on which we focused in this article – is not the only way persons with hysteria try to compensate for their lack of being. Other strategies include defense mechanisms such as repression, conversion, regression, and acting out. Second, seduction is a compensation strategy we find at work in other conditions including, for instance, narcissistic personality, although with relevant differences that we will explain shortly. We want to highlight the centrality of seduction and its typical characteristic in the world of the hysteric person. Last but not least, a lack of being, for which seduction is possibly compensating, is not specific to hysteria. As is well known, a characteristic experience of narcissistic people is a kind of lack of being that consists of a feeling of inner emptiness that requires a recurrent infusion of external confirmations about one’s own importance and value. The difference between hysteria and narcissism, we suggest, is the different ways of coping with a lack of being. Whereas persons with hysteria compensate *via* seduction and centrality to keep the other available and obtain recognition, narcissistic persons strive to attain wealth, beauty, power, and fame to compensate for their lack of being. The imperatives of narcissistic persons, in our age, are production, utility, and optimization (Stanghellini, 2022). Being is equivalent to producing by adapting to existence and by optimizing it. “Utility” means that every action must be directed toward a purpose and, therefore, primarily to production. “Optimization” means that anything that costs more than it produces is a dead branch to be cut. Every aspect of the narcissistic person’s existence is managed as a performance, the aim of which is to compensate for one’s feeling of insufficiency and inferiority, live up to others, and surpass them.

This strategy to get recognition, and the personal values on which it is based, is radically different from the one adopted by persons with hysteria, ie., seduction to keep the other available in an attempt to compensate for lack of being. We explored the implications of this lack of being in the world of persons with hysteria. Its origins lay beyond the scope of the article.

## Data availability statement

The original contributions presented in the study are included in the article/supplementary material, further inquiries can be directed to the corresponding author/s.

## Author contributions

MM wrote the first draft and each section of the manuscript, contributed to the final writing and revision of the manuscript, and read and approved the submitted version. GS contributed to the final writing and revision of the manuscript, and read and approved the submitted version. MS, SM, and VU contributed to the bibliographic research and literature review, and approved the submitted version. All authors contributed to the article and approved the submitted version.

## Funding

The publication of this article was financially supported by “Wellcome Trust Award” (code: 223452/Z/21/Z). Prof Matthew Broome Grant Programme “Discretionary Award – C&S” Title: Renewing Phenomenological Psychopathology University of Birmingham - West Midlands (UK).

## Conflict of interest

The authors declare that the research was conducted in the absence of any commercial or financial relationships that could be construed as a potential conflict of interest.

## Publisher’s note

All claims expressed in this article are solely those of the authors and do not necessarily represent those of their affiliated organizations, or those of the publisher, the editors and the reviewers. Any product that may be evaluated in this article, or claim that may be made by its manufacturer, is not guaranteed or endorsed by the publisher.
